# Vertebrate DM domain proteins bind similar DNA sequences and can heterodimerize on DNA

**DOI:** 10.1186/1471-2199-8-58

**Published:** 2007-07-02

**Authors:** Mark W Murphy, David Zarkower, Vivian J Bardwell

**Affiliations:** 1 Dept. of Genetics, Cell Biology, and Development, 6-160 Church St. SE, University of Minnesota, Minneapolis, MN, 55455, USA

## Abstract

**Background:**

The DM domain is a zinc finger-like DNA binding motif first identified in the sexual regulatory proteins Doublesex (DSX) and MAB-3, and is widely conserved among metazoans. DM domain proteins regulate sexual differentiation in at least three phyla and also control other aspects of development, including vertebrate segmentation. Most DM domain proteins share little similarity outside the DM domain. DSX and MAB-3 bind partially overlapping DNA sequences, and DSX has been shown to interact with DNA via the minor groove without inducing DNA bending. DSX and MAB-3 exhibit unusually high DNA sequence specificity relative to other minor groove binding proteins. No detailed analysis of DNA binding by the seven vertebrate DM domain proteins, DMRT1-DMRT7 has been reported, and thus it is unknown whether they recognize similar or diverse DNA sequences.

**Results:**

We used a random oligonucleotide in vitro selection method to determine DNA binding sites for six of the seven proteins. These proteins selected sites resembling that of DSX despite differences in the sequence of the DM domain recognition helix, but they varied in binding efficiency and in preferences for particular nucleotides, and some behaved anomalously in gel mobility shift assays. DMRT1 protein from mouse testis extracts binds the sequence we determined, and the DMRT proteins can bind their in vitro-defined sites in transfected cells. We also find that some DMRT proteins can bind DNA as heterodimers.

**Conclusion:**

Our results suggest that target gene specificity of the DMRT proteins does not derive exclusively from major differences in DNA binding specificity. Instead target specificity may come from more subtle differences in DNA binding preference between different homodimers, together with differences in binding specificity between homodimers versus heterodimers.

## Background

Sexual reproduction is widespread among metazoans, and hence sexual dimorphisms of anatomy, physiology and behavior that promote efficient reproduction also occur widely. Despite the broad occurrence of sexual dimorphism, most regulators of sex-specific development that have been described appear to function in relatively restricted groups of animals, suggesting that their involvement in this process either is evolutionarily recent or has not been widely maintained. Transcription factors containing the DM domain are an exception, with family members thus far shown to control sexual development in at least three metazoan phyla (insects, nematodes, and vertebrates) [1,2]. In *Drosophila*, the *Doublesex *(*Dsx*) gene controls most aspects of sexual differentiation, functioning in both sexes via sex-specific protein isoforms generated by alternative splicing [3,4]. In *C. elegans*, *mab-3 *[5,6] and *mab-23 *[7] direct aspects of male differentiation. The related gene *Dmrt1 *is required for testicular differentiation in mice [8] and is sex-specifically expressed in other vertebrates with varied sex determination mechanisms [9-11]. In humans, hemizygosity in the chromosomal region containing *DMRT1*, *DMRT3 *and *DMRT2 *(9p24.3) is associated with human testicular dysgenesis and XY male-to-female sex reversal [12-16]. DM domain proteins also may play a role in human cancer: amplification and over-expression of *DMRT1 *is associated with spermatocytic seminoma [17]. The role of this gene family is not limited to sexual differentiation, however: *Dmrt2 *is a critical regulator of vertebrate segmentation [18-20], and *Dmrt4 *has been implicated in olfactory placode neurogenesis in amphibians [21] as well as in ovarian folliculogenesis in mice [22].

The DM domain is an intertwined zinc finger-like DNA binding module [23] first identified in *Dsx *[24] and subsequently found in *mab-3 *[25]. *Dsx *and *mab-3 *control several analogous sexual dimorphisms and the male-specific isoform of DSX (DSX-M) can substitute for MAB-3 in the developing *C. elegans *male nervous system [6,25,26]. The ability of DSX-M to regulate at least one MAB-3 target gene implies that the two proteins must be able to bind a similar DNA sequence. Indeed, in vitro determinations of binding site preference and identification of in vivo binding sites for the two proteins confirm that they bind distinct but overlapping sequences [24,26,27]. The ability of DSX and MAB-3 to bind the same site in vivo is remarkable given the differences in domain organization between the proteins. DSX has a single DM domain and binds DNA as a dimer [28-30], whereas MAB-3 has tandem DM domains, both of which are required for function [25], and binds an asymmetric site, probably as a monomer [26].

The DM domain is notable not only for its apparently ancient and conserved role in regulating sexual differentiation, but also for its unusual structure and mode of DNA interaction. The solution structure of a DSX DM domain peptide was determined and found to be an intertwined module coordinating two zinc atoms, unrelated to structures of other zinc finger motifs [23]. Moreover, base substitution experiments indicate that the DM domain differs from other zinc finger motifs in interacting with DNA primarily via the minor rather than the major groove [23]. Mutational analysis suggests that the DM domain interacts with DNA via the zinc binding module and a C-terminal tail that mediates high-affinity DNA recognition [31]. Structural studies suggest that the C-terminal tail is a nascent alpha helix that becomes ordered upon DNA binding and may widen the minor groove without bending DNA [23]. This mode of interaction with the minor groove is unusual and may be analogous to that of the T box motif [32].

Minor groove binding by DM domain proteins appears to permit them to bind DNA on sites overlapping those of major groove binding proteins. The best studied example is that of DSX binding to an enhancer site in the *Yp1 *yolk protein gene, which contains a binding site for an unidentified bZip protein. DSX-F cooperates with this protein to activate *Yp1 *transcription in females, while DSX-M is thought to antagonize this protein, helping ensure that *Yp1 *is not transcribed in males [33]. The mechanisms involved in these functional interactions are unknown. DSX-M has a larger C-terminal domain than DSX-F, so it has been suggested that DSX-F forms an activating complex with the bZIP and perhaps other proteins, whereas DSX-M may physically occlude binding by the bZIP protein [33]. Interference by DM domain proteins with overlapping transcriptional activators may be a conserved feature: mutational analysis of a regulatory site in the *C. elegans ref-1 *gene indicates that MAB-3 represses transcription of *ref-1 *in part by antagonizing activation by an unknown protein that binds an overlapping site [27].

All higher metazoan species examined have multiple DM domain genes, for example four in *Drosophila*, eleven in *C. elegans*, and seven in mammals. How DM domain proteins achieve specificity for target genes is unknown, but could derive from differences in DNA binding specificity, expression pattern, covalent modifications, interaction with cofactors, or a combination of these. DNA binding specificity has been reported for DSX and MAB-3, as described above. Although these proteins bind related sites, their preferred DNA sequences are sufficiently different that oligonucleotides can readily be designed to bind one but not the other [26]. This is not surprising given the structural differences between DSX and MAB-3, but most DM domain proteins, like DSX, have a single DM domain and thus may bind sites more closely related to that of DSX. A synthetic DMRT1 DM domain peptide has been shown to bind a DSX site, albeit with relatively low affinity [23]. However, no detailed analysis of binding specificity has been performed for any vertebrate DM domain protein.

Here we have investigated DNA binding by the mammalian DM domain proteins. We used an in vitro random oligonucleotide selection method to determine the preferred DNA binding sites of six of the seven mouse DM domain proteins, as well as human DMRT1. We find that these proteins select very similar DNA sequences despite differences in the primary sequence of the DM domain. We also find that DM domain proteins can heterodimerize on DNA, demonstrating that combinatorial regulation of individual target sites by these proteins is possible. Our results indicate that the target site specificity of this important family of regulatory proteins is unlikely to derive entirely from innate DNA binding specificity.

## Results

### DNA binding specificity of DMRT1

**Figure 1:**
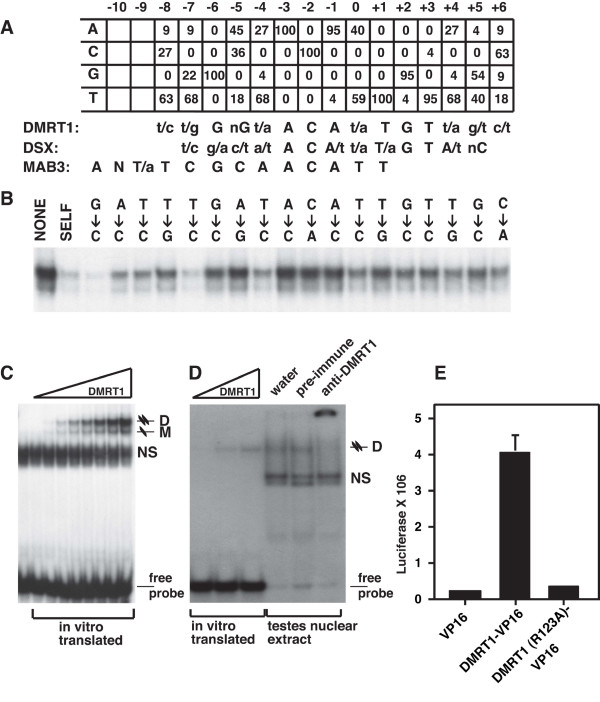
**DNA binding specificity of DMRT1**. (A) Summary of binding site selection. Top panel shows percentage of occurrence of each nucleotide at each position of 22 selected oligonucleotides. (At some positions percentages do not total 100, due to rounding to nearest integer.) Below the selection matrix is a comparison of the DMRT1 binding consensus to those of DSX [29] and MAB-3 [26]. (B) Test of DMRT1 DNA binding specificity. Competitive gel mobility shift assay in which labeled dsDNA probe matching DMRT1 binding consensus is competed with 40-fold excess of unlabelled dsDNA, either unaltered ("SELF") or containing the indicated sequence changes (positions align with those shown in panel A). Only the shifted DMRT1/DNA complex is shown. Darker bands indicate weaker competition. (C) Preferential binding to DNA by DMRT1 as a dimer. Gel mobility shift using increasing amounts of in vitro translated DMRT1 protein to shift a constant amount of labeled dsDNA. Positions of complexes containing monomer ("M") or dimer ("D") of DMRT1 are indicated. (D) DNA binding by DMRT1 from mouse testis. Gel mobility shift using in vitro translated DMRT1 (left) or mouse testis nuclear extract (right) to shift labeled dsDNA containing DMRT1 binding consensus. Addition of water or pre-immune rabbit serum does not affect shifted band, but addition of anti-DMRT1 immune serum [8] super-shifts the band into the gel well. (E) DMRT1 DNA recognition in cultured cells. Luciferase assay results shown for HEK293 cells transfected with expression vectors encoding only VP16, encoding DMRT1-VP16, or encoding DMRT1-VP16 with a missense mutation in the DM domain (R123A). Cells were co-transfected with a reporter plasmid containing four DMRT1 binding sites. Values shown are average of two experiments, with error bar indicating standard error of mean.

**Figure 2:**
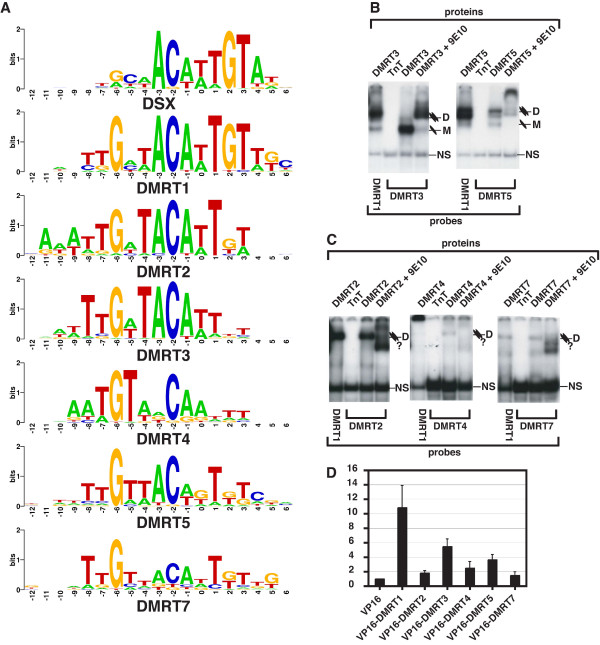
**DNA binding specificity of other DMRT proteins**. (A) Summary of in vitro random oligonucleotide binding site selections for DMRT1, 2, 3, 4, 5, and 7. Sequence preference for DSX is from Yi et al., 1999 [26], reanalyzed using the Gibbs Motif Sampler program [35, 36]. Sequence preferences are displayed using the WebLogo program [44, 45] to indicate preferences at each position, with size of letter indicating information content of each nucleotide (bits). The selected sequences from which each consensus was derived are shown in Supplemental Figure 1. (B,C) Gel mobility shift analysis. (B) DMRT3 and DMRT5. (C) DMRT2, DMRT4, DMRT7. Labeled probes used are indicated below each panel, and proteins and antibodies present in binding reactions are indicated above. Mobility of dimer and monomer complexes with DNA are indicated for each protein ("M" or "D"); "NS" indicates non-specific complexes. Exposure time for panel C is 28-fold longer than for panel B. (D) DNA recognition by DMRT proteins in cultured cells. Transactivation by VP16 fusion proteins in HEK293 cells transfected with expression vectors encoding the indicated DMRT-VP16 fusions and with the pGL3-Promoter reporter plasmid containing a single DMRT consensus sequence upstream of the luciferase coding sequence. Values shown are average of two experiments, with error bar indicating standard error of the mean.

To define high-affinity DNA binding sites for mouse DMRT1, we used a selection method in which bound oligonucleotides are isolated from a large pool of random double-stranded oligonucleotides by immunoprecipitation of protein/DNA complexes [34]. Recovered DNA is amplified by PCR using flanking primers and reselected to further enrich for high-affinity binding sites. Full-length DMRT1 protein was made by in vitro transcription/translation and contained an amino-terminal Myc epitope tag. After three rounds of DNA binding and immunoprecipitation, a final selection was performed using a gel mobility shift, and the shifted oligonucleotides were cloned and sequenced. DNA sequences of the selected oligonucleotides were compared using the Gibbs Motif Sampler algorithm [35,36] to identify potential binding site preferences (Methods). This revealed a consensus sequence (Figure 1A) closely resembling that bound by DSX [29] and also similar to the site bound by MAB-3 [26]. Like the previously determined DSX consensus, the DMRT1 site contains an inverted repeat of six nucleotides flanking a central A/T basepair (position 0). The most obvious difference between the sites is that DMRT1 always selected a G at position -6 whereas DSX lacked a strong preference at this position (see Figure 2A and Additional file 1). Competitive gel mobility shift assays (Figure 1B, described below; and data not shown) confirmed that this represents a real difference in binding preference between the two proteins, as introducing a C at this position severely compromised binding by DMRT1 but not DSX. The DMRT1 site is partially asymmetric: selection of preferred nucleotides extends further on the left side of the inverted repeat than on the right side (as oriented in Figure 1A). An analogous site selection using human DMRT1 protein identified a nearly identical binding site (not shown).

To verify that DMRT1 binds to the selected consensus, we used a competitive gel mobility shift assay (Figure 1B). We made a set of unlabeled competitor double-stranded oligonucleotides, each with a single position of the binding site changed to the least-preferred nucleotide or one that was never selected. We assayed the ability of these altered sites to compete for DMRT1 binding against a labeled probe that conforms to the binding site consensus. At the competitor concentrations used, a competitor with an unaltered binding site ("self" in Figure 1B) strongly reduces binding to the labeled probe. Therefore, a strong shifted band indicates that a change in that nucleotide eliminates effective competition by the unlabeled DNA. All single base changes to nucleotides that were never selected reduced binding, with the strongest affects within the core 13 bp inverted repeat sequence. One position outside the 13 bp core (-8) was strongly preferred, and changing this to a non-selected nucleotide strongly reduced binding. Based on these results, the binding consensus matrix appears to sensitively reflect the binding preference of DMRT1, at least in vitro. The close similarity of the DMRT1 site to a DSX site is consistent with the previous observation that the DMRT1 DM domain can weakly bind to a DSX site [23]. Like DSX [23,29], DMRT1 appears to bind DNA as a dimer. This is supported by heterodimerization experiments using proteins of different sizes (see below). Even at limiting protein concentrations DMRT1 preferentially forms dimers rather than monomers on DNA (Figure 1C), indicating that binding of the first molecule facilitates binding of the second.

We next tested whether the site defined using in vitro translated protein is bound by endogenous DMRT1 from testis nuclear extracts. Testis extracts shift a DMRT1 site probe to the same electrophoretic mobility as the complex formed with in vitro translated DMRT1 (Figure 1D). This complex is super-shifted by a DMRT1 antibody, confirming the presence of DMRT1 in the complex. We also tested human DMRT1 binding in transfected cells using a fusion of DMRT1 to the VP16 strong transcriptional activation domain. (DMRT1 lacking VP16 had no specific effect on reporter expression in this cell type.) HEK293 cells transfected with a luciferase reporter containing a single DMRT1 binding site show strong transcriptional activation when co-transfected with a vector expressing DMRT1-VP16, but not when transfected with a vector expressing VP16 alone (Figure 1E). Previous work identified several amino acid residues in the DSX DM domain that are critical for DNA binding and cause loss-of-function mutant phenotypes [29,31]. Mutating DMRT1-VP16 at one of these positions (R123A; equivalent to R91 in DSX) nearly abolishes reporter activation, even when the mutant protein is expressed at a higher level than the wild type protein (Figure 1E and data not shown).

### Binding specificity of other DMRT proteins

The preceding results show that the in vitro binding site selection strategy we used for DMRT1 can identify a high-affinity binding site capable of interaction in vitro and in cultured cells. To compare DNA binding specificity among DM domain proteins, we next selected binding sites for the other six mouse DMRT proteins, and were able to define binding site consensus sequences for all except DMRT6. The other five proteins selected sites that can be aligned with DMRT1 and DSX [26,29] sites, with the closest similarity on one side of the 13 bp core DMRT1 consensus sequence (Figure 2A and Additional file 1). Consistent with this similarity, all five proteins can shift a DMRT1 consensus site to varying degrees in a gel mobility assay (Figure 2B,C).

The proteins differed greatly in how readily they selected oligonucleotides containing binding sites and in their behavior in the gel mobility shift assay. DMRT5 selected a consensus sequence after two rounds of immunoprecipitation followed by a gel shift, while DMRT3, like DMRT1, required three rounds. In contrast, DMRT2 and DMRT7 required five rounds of immunoprecipitation prior to the gel shift. DMRT4 did not show a shifted band in the gel shift assay even after five rounds of immunoprecipitation, and thus its binding preference was determined by sequencing oligonucleotide pools after the fifth round of immunoprecipitation. DMRT3 and DMRT5 efficiently formed protein/DNA complexes that could be supershifted with the 9E10 anti-Myc antibody (Figure 2B). Although DMRT5 selected a consensus in just two rounds, it bound a site conforming to this consensus less efficiently than it bound a DMRT1 site. This indicates that the DMRT5 oligonucleotide pool had not fully evolved toward high affinity sites after two rounds. DMRT2 and DMRT7 also formed specific protein/DNA complexes with either their cognate sites or a DMRT1 site, but DNA binding by these proteins was much less efficient than that of DMRT1 (Figure 2C; note 28-fold longer exposure time relative to Figure 2B). DMRT4 weakly shifted a DMRT4 probe, but on a DMRT1 site it formed complexes that failed to enter the gel (Figure 2C). As lysates programmed with other DMRT proteins did not form these complexes, we conclude that they contain DMRT4, possibly in a high-order complex or aggregate.

It is unclear why the DMRT2, DMRT4, and DMRT7 proteins behaved differently from the others, as all were expressed at similar levels (data not shown) and selections were performed in parallel under identical conditions. Possibly this reflects differences among the DMRT proteins in optimal DNA binding conditions or in their mode of DNA interaction, as appears to be the case with DMRT4 on a DMRT1 site. Despite these differences in binding behavior, all six proteins when fused to VP16 can activate transcription from their selected sites in transfected HEK293 cells (Figure 2D). The magnitude of activation varied between experiments, but DMRT1 consistently showed the strongest activation, followed by DMRT3 and DMRT5, with DMRT2, DMRT4, and DMRT7 showing the weakest activation.

DMRT1, DMRT3, and DMRT5 all selected sites readily and bound efficiently. Although similar, the binding sites selected were not identical. Also, DMRT3 selected a site to which it primarily binds as a monomer (Figure 2B), suggesting that this protein may have higher monomeric DNA binding affinity than the other DMRT proteins. This does not represent an absolute preference for monomer binding, however, as DMRT3 preferentially binds as a dimer to a DMRT1 site (Figure 2B).

**Figure 3:**
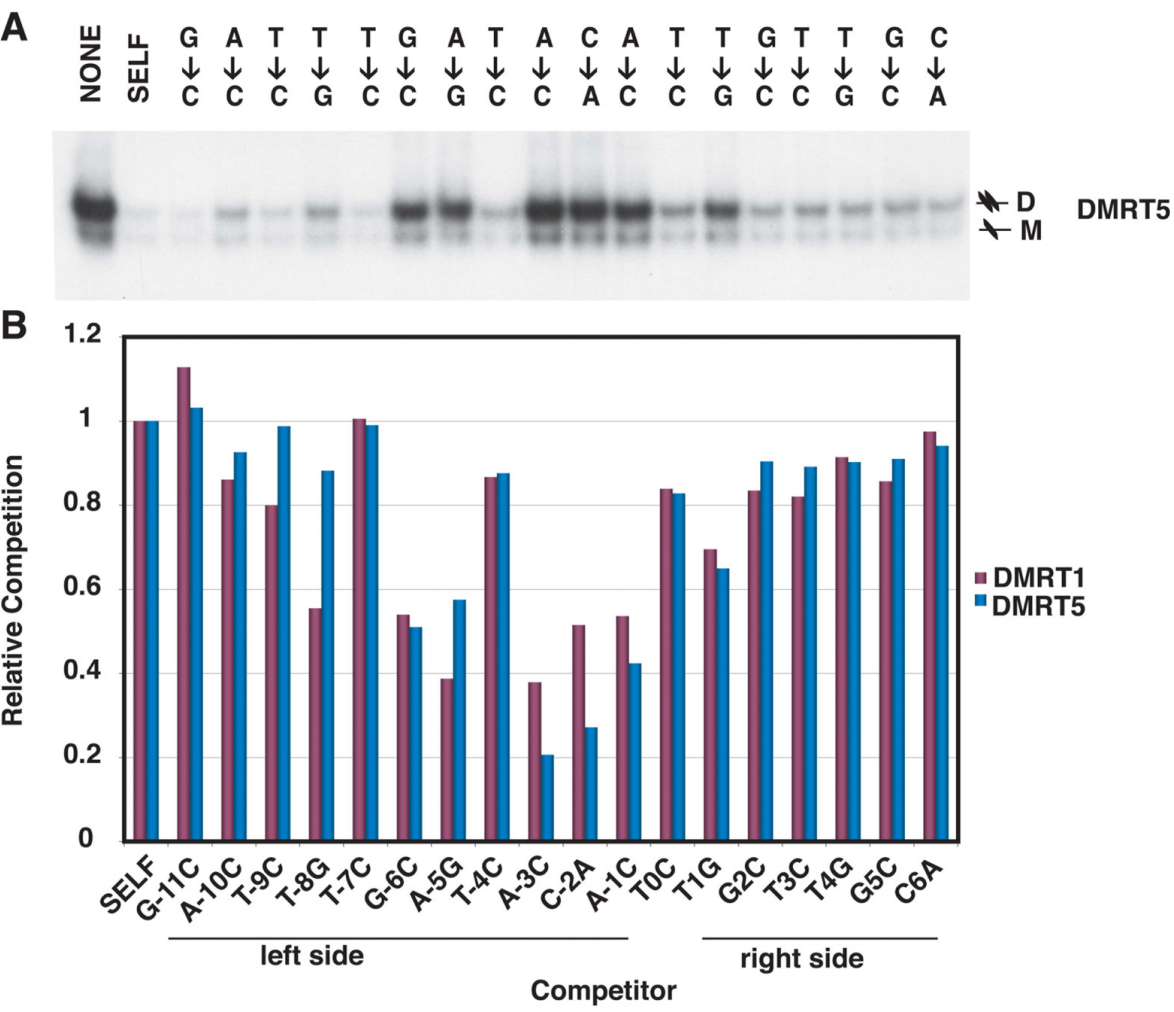
**Comparison of DNA binding preferences of DMRT1 and DMRT5**. Top panel: competitive gel mobility shift using the same probe and competitors as in Figure 1A, testing binding by in vitro translated DMRT5. Positions of monomeric and dimeric complexes are indicated ("M" or "D"). Bottom panel: effectiveness of competitor DNAs. Quantitation of data from top panel and Figure 1A, with competition by perfect DMRT1 site ("SELF") set at 1.0.

We next investigated the extent of similarity in DNA binding specificity between DMRT1 and DMRT5. To do this, we first repeated the competitive gel mobility shift assay of Figure 1A, this time testing binding of DMRT5 to a labeled DMRT1 site. We asked whether the same set of unlabeled competitor DNAs had the same or different effects on binding of DMRT1 (Figure 1A) and DMRT5 (Figure 3A). Quantitation of the gel assays in Figures 1A and 3A is shown graphically in Figure 3B, which indicates how well each altered oligonucleotide competes, relative to an unaltered oligonucleotide. It is apparent from this analysis that base changes in the left half of the core binding site (-1 to -6, except -4) affect DNA binding of both proteins more severely than those on the right side (+1 to +6). This suggests a more critical role for the left side in DNA binding by both proteins. This analysis also highlights one position (-8) that appears to be particularly important in discriminating between binding by the two proteins. DMRT1 never selected a G at -8 and does not tolerate one. In contrast, DMRT5 occasionally selected a G at -8 (6% of sites) and can tolerate one. This confirms that the preferred binding sites of DMRT1 and DMRT5, while similar, are not identical, suggesting that they do not regulate identical sets of target genes.

**Figure 4:**
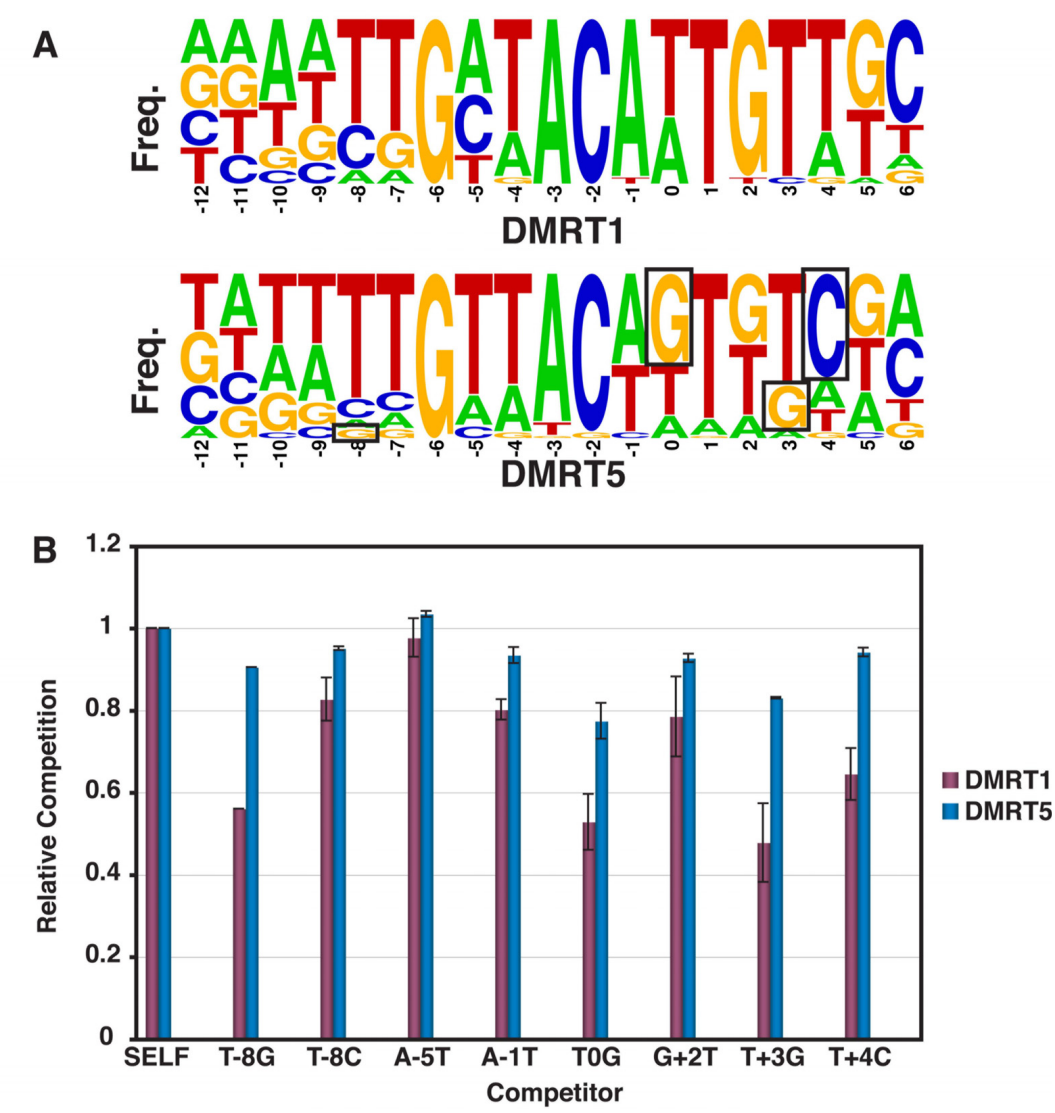
**Nucleotides that discriminate between binding by DMRT1 and DMRT5**. (A) Comparison of sites selected by DMRT1 and DMRT5. WebLogo representation of frequency of occurrence of each base at each position in oligonucleotides selected by DMRT1 and DMRT5. Boxed residues are those selected by DMRT5 and not by DMRT1. (B) Competitive gel mobility shift assay results. Ability of unlabeled DNAs with the indicated changes to compete for binding with a labeled DMRT1 site DNA is plotted for each protein. Competition by an unaltered ("SELF") unlabelled DNA is set at 1.0 for each protein.

The observation that the nucleotide identity at position -8 discriminates between binding of DMRT1 versus DMRT5 led us to investigate other positions at which nucleotide identity might be discriminatory for these two proteins. We selected six additional positions at which there was a significant difference in nucleotide preferences between DMRT1 and DMRT5, and tested the effect of changes at these positions on DNA binding, assayed by competitive gel shift analysis (Figure 4A, B). We focused on positions at which a particular nucleotide was selected more frequently by DMRT5 than by DMRT1. When a particular nucleotide was never selected by DMRT1, making that change had a stronger effect on binding by DMRT1 than on binding by DMRT5 (Figure 4B, positions -8G, 0G, +3G, and +4C). By contrast, when a particular nucleotide was occasionally selected by DMRT1, making that change had a modest effect on binding by both proteins. For example, at position -8, a C, which is occasionally selected by both proteins, had little or no effect on binding by either protein. From these results we conclude that the DMRT1 and DMRT5 DNA binding preferences are very similar, but that specific nucleotides can discriminate between binding of the two proteins. This systematic comparison also indicates that the binding site selection is an accurate indication of DNA site preference for these proteins, at least in vitro.

**Figure 5:**
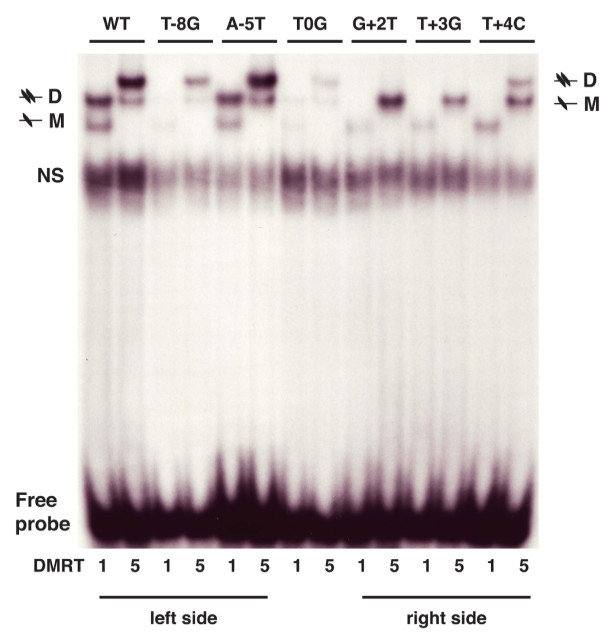
**Monomer versus dimer binding by DMRT1 and DMRT5**. Binding of DMRT1 and DMRT5 to labeled probes with single base changes from the DMRT1 consensus. In vitro translated protein used is indicated, as is position of monomeric and dimeric complexes for each protein.

Based on the site selection data, in which the left side of each site was more strongly selected by DMRT1 and DMRT5 than the right side, we hypothesized that protein dimer formation might occur via initial binding to the left side of the site, followed by recruitment of a second monomer to the right side. A prediction of this model is that sequence changes on the left side of the site that reduce protein binding should not block dimer formation, whereas changes to the right side should primarily reduce dimer formation without strongly affecting monomer binding. We tested six sequence changes, and found that their effects on binding by both proteins are consistent with this model (Figure 5).

### Heterodimerization of DMRT proteins

As described above, the DMRT proteins bind very similar inverted repeat sequences. DSX has been shown to bind its site cooperatively as a dimer. Dimerization motifs have been mapped to the DM domain and to a C terminal domain [28,29]. The DMRT proteins also appear to bind as dimers and may in some cases be co-expressed [37], raising the possibility of heterodimerization between DMRT proteins. We therefore tested whether the DMRT proteins can form heterodimers on DNA.

**Figure 6:**
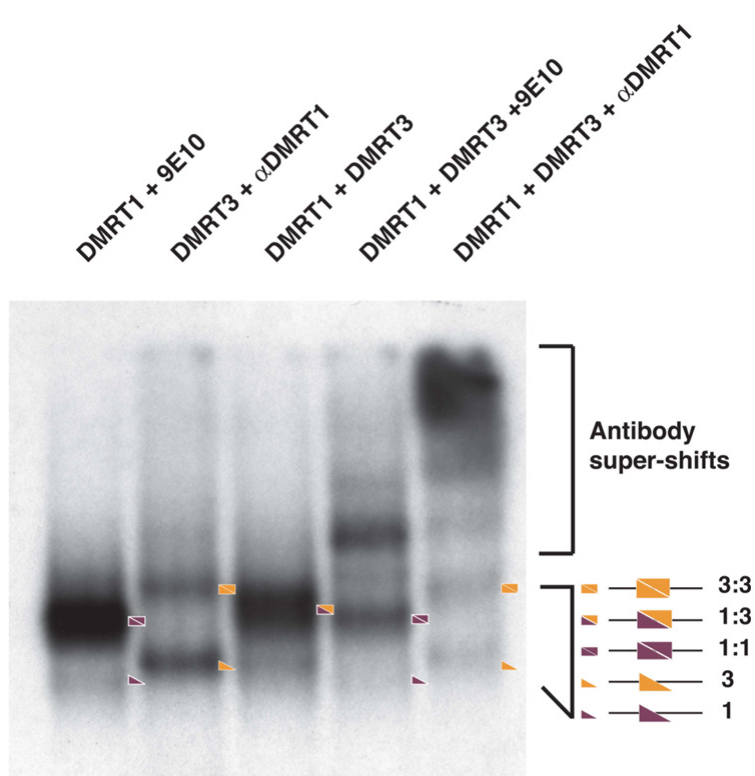
**Heterodimer formation between DMRT1 and DMRT3**. Gel mobility shifts using labeled DMRT1 consensus probe DNA and indicated in vitro translated proteins. Positions of monomeric, and homo- and heterodimeric complexes with DNA are indicated (maroon triangles indicate DMRT1 and gold triangles indicate DMRT3). DMRT3 protein is tagged with a myc epitope recognized by 9E10 monoclonal antibody. As shown in first two lanes, 9E10 does not affect complex formed by DMRT1, and anti-DMRT1 antibody does not affect complex formed by tagged DMRT3.

We examined heterodimerization by gel mobility shift analysis. We tested binding of DMRT1 to its consensus site, either alone or in the presence of a Myc-tagged DMRT protein. To determine the composition of the resulting DNA/protein complexes, we used either a DMRT1 or an anti-Myc (9E10) antibody. We focused on DMRT3 and DMRT5 as potential dimer partners, as they bind efficiently to a DMRT1 site. When DMRT1 and DMRT3 were present in the same binding reaction, we observed a complex intermediate in electrophoretic mobility between the homodimeric complexes formed by each of the proteins alone (Figure 6). The putative heterodimer complex was supershifted by both DMRT1 and anti-Myc antibodies, demonstrating that it contains DMRT1 and DMRT3. DMRT1 and DMRT5 also formed heterodimeric complexes with DNA (see Additional file 2). We therefore conclude that DMRT1 can form heterodimers on DNA with DMRT3 and with DMRT5, at least in vitro.

## Discussion

We have used an in vitro DNA binding site selection procedure to define the binding site preferences for six of the seven mouse DMRT proteins. The main conclusions of the work presented here are that the DMRT proteins select similar DNA binding consensus sites, which resemble that of DSX, and that some DMRT proteins can heterodimerize on DNA. The sites selected by the different proteins were not identical, however, and we observed qualitative and quantitative differences in DNA binding behavior in gel mobility shift assays. These differences may have implications for DNA binding in vivo, as discussed below.

The DM domain is bipartite, comprised of an amino-terminal zinc-binding module and a more variable carboxy-terminal domain that has been proposed to act as a recognition helix, contributing to DNA binding affinity and specificity [23,31]. Mutagenesis studies have identified a number of residues in both portions of the DSX DM domain that are critical for binding to its consensus site [38]. The DMRT proteins have recognition helices that differ from that of DSX at three of these critical positions. Despite these differences, however, most of the DMRT proteins select a site similar to that of DSX, and indeed several can efficiently bind a DSX site in a gel shift assay (data not shown). The similarity in DNA binding is surprising, given that all of the DMRT proteins have an amino acid change that eliminates efficient DNA binding by DSX (D78E in DSX) [38]. The ability of the DSX and DMRT DM domains, despite their different recognition helices, to bind similar sequences underscores the need for a DM domain/DNA co-structure to help define how specific DM domain residues contribute to DNA affinity and to DNA sequence specificity. It also will be interesting to determine the preferred sites of more highly divergent DM domains such as those found in *C. elegans *and see whether they also bind sites similar to that of DSX.

The sites selected by the DMRT proteins were very similar and most of the DMRT proteins were able to shift a DMRT1 site in the gel shift assay. However there were distinct sequence preferences at some positions. We tested the significance of the differences in sites selected by DMRT1 and DMRT5, and confirmed that several differentially selected nucleotides do have differential effects on binding by the two proteins in a gel shift assay (Figures 3 and 4). Based on these comparisons, it therefore appears that there are distinct binding preferences among the DMRT proteins, at least in vitro. The extent to which such differences in sequence preference also exist in vivo will require the future identification and characterization of DMRT target genes.

As described earlier, we were able to select bound DNA in solution using all of the DMRT proteins except DMRT6, but there were differences in the behavior of several proteins in the gel mobility shift assay that may be significant. DMRT2 and DMRT7 selected sites resembling those of the other DMRT proteins and DSX, indicating similar specificity, but exhibited less efficient DNA binding in the gel mobility shift assay, suggesting lower affinity for DNA. Although similar amounts of each protein were tested, we cannot exclude the alternative possibility that some of the proteins folded inefficiently and thus were less active. In contrast to the other proteins tested, DMRT4 formed a protein/DNA complex on a DMRT1 site that was excluded from the gel. There are several possible explanations for these differences in DNA binding behavior, which are not mutually exclusive. First, some of the proteins may form structurally distinct protein/DNA complexes, and this may be reflected in their behavior in the gel shift assay. For example, DMRT4 may form higher-order multimers on DNA. Second, efficient binding of proteins like DMRT2 and DMRT7 may require covalent modifications that were missing from the in vitro translated proteins. Third, some of the proteins may normally bind DNA in conjunction with one or more partner proteins (DM domain or otherwise) that were missing from the in vitro assay. A possible example is the efficient binding of DMRT3 as a heterodimer with DMRT1 (see below).

The DMRT protein binding sites, like that of DSX, comprise a punctuated palindrome flanking a single A/T base pair, and we found that DMRT1 and DMRT5 bind to this site as dimers. The site is not fully symmetrical, however, and there was stronger selection of DNA sequence on one side of most sites. Extensive sequence substitutions indicated that the side with the stronger selection is required for efficient DNA binding of both monomers and dimers, whereas the other side is primarily needed to allow dimer binding. Our data suggest a model in which the left side of the binding site is recognized first by a DMRT monomer. Once the first DMRT molecule has bound to the DNA, it presents a surface that in combination with the right side of the DNA site creates a high affinity binding site, which, allows a second DMRT molecule to efficiently bind. This model is supported by the base substitution experiments in Figure 4C.

We found that DMRT1 can heterodimerize on DNA with DMRT3 and DMRT5, raising the possibility of combinatorial gene regulation by DMRT proteins. It is particularly striking that DMRT3 preferentially binds as a heterodimer in vitro. Comprehensive expression analysis has not been reported for most *Dmrt *genes, but it is clear that at least some overlapping expression occurs. For example, *Dmrt1 *and *Dmrt3 *are coexpressed in spermatogonia and Sertoli cells in the adult testis (unpublished data and the Mammalian Reproductive Genetics  Database). Because the DMRT proteins vary in sequence preference outside the core consensus (Figure 2A), heterodimer formation has the potential to significantly increase selectivity for specific target sites. In addition, because the carboxy termini of the DMRT proteins are highly variable, it is possible that heterodimers can interact with a different spectrum of transcriptional regulatory partner proteins than do homodimers. It will be of interest in the future to better define the overlap of DM domain protein expression in mammals and other animals and to test the effect of heterodimerization on DNA binding specificity.

In contrast with the other DMRT proteins, DMRT6 did not select binding sites, and we were unable to detect binding of DMRT6 to a DMRT1 site (not shown). The other DMRT proteins are identical across 14 positions in the recognition helix (13 in DMRT7), whereas DMRT6 differs at six of these 14 positions. One of these variant residues is an arginine in the other DMRT proteins but a threonine in DMRT6, and is the site of a known intersex mutation in DSX (R91Q). Additionally, and perhaps more significantly, within the zinc binding module, a conserved lysine residue that in DSX has been proposed to form an important salt bridge to the DNA backbone (K60), instead is substituted with an alanine in DMRT6. We therefore suggest that DMRT6 may not directly bind DNA. We cannot, however, exclude the possibility that our in vitro conditions are incompatible with DNA binding by DMRT6.

An important question is how closely the DNA binding sites we have determined in vitro resemble those in vivo. Particularly for the proteins that did not exhibit efficient binding as homodimers, the sites occupied in vivo may only partially resemble those determined in vitro. Ultimately this question must be answered by identifying the in vivo targets of the DMRT proteins. In flies and worms DM domain proteins have been shown to use in vivo sites that closely mirror their in vitro binding preferences: DSX regulates transcription of the yolk protein gene *Yp1 *in flies and MAB-3 regulates transcription of the yolk protein gene *vit-2 *and the antineural bHLH gene *ref-1 *in worms and this requires sites closely matching those preferred in vitro [26,27,39]. These results suggest that at least some of the DMRT proteins are likely to regulate their in vivo targets via sites similar to the ones we have defined.

## Conclusion

Selection of DNA binding sites from random oligonucleotide pools demonstrates that mammalian DMRT proteins prefer sequences similar to those bound by DSX of Drosophila. Like DSX, the DMRT proteins have higher sequence specificity than most minor groove DNA binding proteins. The preferred DMRT binding sites differ at a few positions, and the differences can affect binding efficiency. However, in some cases different DMRT proteins can efficiently bind to the same sequence, so it is unlikely that in vivo target gene specificity is governed only by binding site sequences. DMRT proteins can heterodimerize on DNA and in some situations bind more efficiently as heterodimers than as homodimers. From this we conclude that heterodimer formation may contribute to transcriptional regulation by DMRT proteins.

## Methods

### DNA binding site selection

DMRT protein coding sequences (except DMRT4) were cloned into a derivative of T7βplink [40] that places a 9E10 Myc epitope tag at the amino terminus (pT7 Ntag plink); the DMRT4 coding sequence was inserted into another T7βplink derivative (pT7 plink Ctag) that places the tag at the carboxy terminus. Proteins were produced using the TNT™ Quick Coupled transcription/translation system (Promega) according to the manufacturer's instructions, except that 20 μM ZnSO4 was included. Site selections were carried out essentially as described [34], except that binding reactions contained 10 mM Tris-HCl pH 7.9, 100 mM KCl, 10% glycerol, 5 mM MgCl_2_, 0.075%Triton-X-100, 1 mM DTT, 1 μg BSA, 0.1 μg dI-dC, 75 ng plasmid DNA (as a more complex non-specific competitor), 0.2 ng radiolabeled duplex probe, and 4 μl in vitro translation mix, in a total volume of 20 μl. After co-incubation of the epitope-tagged proteins with the oligonucleotide pools, complexes were recovered using a mMACS™ epitope tag protein isolation kit (Miltenyi Biotec #130-091-123). After each round of immunoprecipitation we tested whether each protein formed shifted bands in the gel mobility shift assay (see below), and cloned and analyzed sites from the upper shifted band (presumed to contain DMRT dimers) from the first round in which shifted bands were apparent. We also confirmed, by antibody supershift, that each shifted band contained the protein of interest.

### Site selection analysis

Sequences of selected oligonucleotides were compared and any duplicates eliminated from the analysis. Sequences were trimmed to retain 5 nucleotides of the primer binding site on each side of the variable sequence. Gibbs Motif Sampler [41] was used to align sequences. In some DMRT selections the binding site in a small subset of sequences overlapped for several base pairs in a consistent way with the fixed primer binding site. These sequences were not included in the final analysis. The consensus sites encompass all nucleotide positions with a Gibbs information content of 0.3 or greater (DSXF 13 bp, DMRT1 15 bp, DMRT2 16 bp, DMRT3 15 bp, DMRT4 13 bp, DMRT5 15 bp, DMRT7 14 bp, see Additional file 1). Fewer than 5% of oligonucleotides contained no discernible site conforming to the preliminary consensus, and these were excluded from the final analysis. Weblogo [42] was used to generate representations of the consensus sequences. To generate comparable Weblogo representations for Figure 2a, sequences flanking the consensus sequence were included (see Additional file 1)

### Gel mobility shift assay

(1RE) 5'-TCGAGATTTGATACATTGTTGC-3' and complement with overhanging XhoI ends were annealed by heating to 80°C for three minutes in 20 mM Hepes-KOH [pH 7.9], 50 mM NaCl, and 6 mM MgCl_2_; slow-cooled to 50°C; held at that temperature for ten minutes; and cooled to room temperature. After annealing and cooling, the duplex oligonucleotides were de-salted through a Sephadex G-50 spin column. The de-salted duplexes were labeled with Klenow DNA polymerase and α [^32^P]-dCTP. Unincorporated nucleotides were removed through a Sephadex G-50 spin column.

DNA binding reactions contained 10 mM Tris-HCl (pH 7.9), 100 mM KCl, 10% glycerol, 5 mM MgCl_2_, 1 mM spermidine, 0.075%Triton-X-100, 1 mM DTT, 1 μg BSA, 0.1 μg dI-dC, 75 ng plasmid DNA (as a more complex non-specific competitor), 0.5 ng radio-labeled duplex probe, and 4 μl in vitro translation mix, in a total volume of 20 μl. Binding reactions were performed on ice for ten minutes and complexes were resolved on a 5% native acrylamide (37.5:1) gel in 0.5× TB. Gels were run at 300 v for 1 hour and 45 minutes at 4°C, then dried and subjected to autoradiography. Quantitation was performed using the Molecular Dynamics PhosphoImager using the Image quant software. For supershift and competition experiments, the antibody or unlabeled competitor (40-fold molar excess) was added at the start of the ice incubation. Gel mobility shifts using testes nuclear extract, contained 4 μl testis extract, 225 ng non-specific competitor, and omitted MgCl_2_.

For Figure 2 the top strand probes were

(1RE) 5'-TCGAGCCCGCAACAATGTTGCAAATC-3',

(2RE) 5'-TCGAGCGATACAATGTATCAATTTGC-3',

(3RE) 5'-TCGAGAACAATGTAACAATTTCGCCC-3',

(4RE) 5'-TCGAGAAAATGTAACAATTTAGCCGC-3',

(5RE) 5'-TCGAGCGCTACTGTTACATTGTCGCC-3',

(7RE) 5'-TCGAGTCTCAACAATGTAACAATTTC-3'

Each of these oligonucleotides was annealed to its complement, with overhanging XhoI ends, and labeled as described above.

### Testis nuclear extracts

Extracts were prepared from sixteen 3.5 week old male mice. Testes were rinsed in phosphate-buffered saline and homogenized in 25 ml of extraction buffer (10 mM Hepes-KOH [pH 7.9], 10 mM KCl, 0.1 mM EDTA, 0.74 mM spermidine, 1 mM DTT, 0.5 mM PMSF, 2 M sucrose) with five strokes of a dounce homogenizer using the B (loose) pestle. The homogenate was spun through a 10 ml cushion of the same solution at 24 k rpm for 30 minutes at 4°C in an SW28 rotor. After removal of the supernatant, recovered nuclei were resuspended in 5 ml of extraction buffer containing 0.42 M NaCl, 5 mM DTT, and protease inhibitors, and stirred at 4°C for one hour. After pelleting at 15 k rpm for 30 minutes in an SS-34 rotor, the supernantant was snap-frozen and stored at -70°C.

### VP16 activation cell based assay

For Figure 2D, coding sequences for each of the DMRT proteins were inserted into pKH68, an EF-1α promoter-driven expression vector [43], generating constructs fusing an SV40 nuclear localization signal, a Myc epitope tag, and the VP16 strong transcriptional activation domain to the N-terminus of the protein. For Figure 1E, coding sequences for human DMRT1, the VP16 domain and a Myc epitope tag were inserted into the T7/CMV promoter-driven pCMX vector such that DMRT1 is N-terminal to the VP16 activation domain and Myc tag. Luciferase reporter plasmids were generated by inserting a single copy (or for Figure 1E, four copies) of the consensus binding sequence for each DMRT protein into the *Xho*I site of the reporter plasmid pGL3-Promoter (Promega). Each VP16 fusion plasmid was cotransfected into HEK293 cells with the appropriate reporter plasmid and a CMV-lacZ plasmid (for normalization of transfection efficiency). Luciferase levels were compared to those of cells co-transfected with each DMRT reporter plasmid and empty pKH68. The top strand primers used to generate the reporter constructs were the same as those used in the Figure 2 gel shift assays.

### Mutagenesis

Mutants were generated using the GeneEditor™ system from Promega according to the manufactures instructions. The primer used to generate the R123A mutant in hDMRT1 was 5'-Phos-CAGGTGGCCCTGAGAGCGCAGCAGGCCCAGGA-3'.

## Authors' contributions

MWM participated in the design of the study, carried out all experiments, participated in analysis of binding site selection data, and helped draft the manuscript. DZ participated in the design of the study and helped draft the manuscript. VJB participated in the design of the study, analyzed binding site selection data, and helped draft the manuscript. All authors together completed and approved the final manuscript.

## Supplementary Material

Additional file 1 Sequences of selected sites. This file includes all of the selected sequences used to determine the described binding consensus sites. Click here for file

Additional file 2 Heterodimer formation between DMRT1 and DMRT5. This file shows that DMRT1 can form heterodimers on DNA with DMRT5. Click here for file

## References

[B1] Zarkower D (2001). Establishing sexual dimorphism: conservation amidst diversity?. Nat Rev Genet.

[B2] Hodgkin J (2002). The remarkable ubiquity of DM domain factors as regulators of sexual phenotype: ancestry or aptitude?. Genes Dev.

[B3] Baker BS, Ridge KA (1980). Sex and the single cell. I. On the action of major loci affecting sex determination in Drosophila melanogaster. Genetics.

[B4] Burtis KC, Baker BS (1989). Drosophila doublesex gene controls somatic sexual differentiation by producing alternatively spliced mRNAs encoding related sex-specific polypeptides. Cell.

[B5] Shen MM, Hodgkin J (1988). mab-3, a gene required for sex-specific yolk protein expression and a male-specific lineage in C. elegans. Cell.

[B6] Yi W, Ross JM, Zarkower D (2000). Mab-3 is a direct tra-1 target gene regulating diverse aspects of C. elegans male sexual development and behavior. Development.

[B7] Lints R, Emmons SW (2002). Regulation of sex-specific differentiation and mating behavior in C. elegans by a new member of the DM domain transcription factor family. Genes Dev.

[B8] Raymond CS, Murphy MW, O'Sullivan MG, Bardwell VJ, Zarkower D (2000). Dmrt1, a gene related to worm and fly sexual regulators, is required for mammalian testis differentiation. Genes Dev.

[B9] Raymond CS, Kettlewell JR, Hirsch B, Bardwell VJ, Zarkower D (1999). Expression of Dmrt1 in the genital ridge of mouse and chicken embryos suggests a role in vertebrate sexual development. Dev Biol.

[B10] Smith CA, McClive PJ, Western PS, Reed KJ, Sinclair AH (1999). Conservation of a sex-determining gene. Nature.

[B11] Kettlewell JR, Raymond CS, Zarkower D (2000). Temperature-dependent expression of turtle Dmrt1 prior to sexual differentiation. Genesis.

[B12] Raymond CS, Parker ED, Kettlewell JR, Brown LG, Page DC, Kusz K, Jaruzelska J, Reinberg Y, Flejter WL, Bardwell VJ, Hirsch B, Zarkower D (1999). A region of human chromosome 9p required for testis development contains two genes related to known sexual regulators. Hum Mol Genet.

[B13] McDonald MT, Flejter W, Sheldon S, Putzi MJ, Gorski JL (1997). XY sex reversal and gonadal dysgenesis due to 9p24 monosomy. Am J Med Genet.

[B14] Veitia R, Nunes M, Brauner R, Doco-Fenzy M, Joanny-Flinois O, Jaubert F, Lortat-Jacob S, Fellous M, McElreavey K (1997). Deletions of distal 9p associated with 46,XY male to female sex reversal: definition of the breakpoints at 9p23.3-p24.1. Genomics.

[B15] Calvari V, Bertini V, De Grandi A, Peverali G, Zuffardi O, Ferguson-Smith M, Knudtzon J, Camerino G, Borsani G, Guioli S (2000). A new submicroscopic deletion that refines the 9p region for sex reversal. Genomics.

[B16] Guioli S, Schmitt K, Critcher R, Bouzyk M, Spurr NK, Ogata T, Hoo JJ, Pinsky L, Gimelli G, Pasztor L, Goodfellow PN (1998). Molecular analysis of 9p deletions associated with XY sex reversal: refining the localization of a sex-determining gene to the tip of the chromosome. Am J Hum Genet.

[B17] Looijenga LH, Hersmus R, Gillis AJ, Pfundt R, Stoop HJ, van Gurp RJ, Veltman J, Beverloo HB, van Drunen E, van Kessel AG, Pera RR, Schneider DT, Summersgill B, Shipley J, McIntyre A, van der Spek P, Schoenmakers E, Oosterhuis JW (2006). Genomic and expression profiling of human spermatocytic seminomas: primary spermatocyte as tumorigenic precursor and DMRT1 as candidate chromosome 9 gene. Cancer Res.

[B18] Seo KW, Wang Y, Kokubo H, Kettlewell JR, Zarkower DA, Johnson RL (2006). Targeted disruption of the DM domain containing transcription factor Dmrt2 reveals an essential role in somite patterning. Dev Biol.

[B19] Saude L, Lourenco R, Goncalves A, Palmeirim I (2005). terra is a left-right asymmetry gene required for left-right synchronization of the segmentation clock. Nat Cell Biol.

[B20] Meng A, Moore B, Tang H, Yuan B, Lin S (1999). A *Drosophila* doublesex-related gene, terra, is involved in somitogenesis in vertebrates. Development.

[B21] Huang X, Hong CS, O'Donnell M, Saint-Jeannet JP (2005). The doublesex-related gene, XDmrt4, is required for neurogenesis in the olfactory system. Proc Natl Acad Sci U S A.

[B22] Balciuniene J, Bardwell VJ, Zarkower D (2006). Mice mutant in the DM domain gene Dmrt4 are viable and fertile but have polyovular follicles.. Mol Cell Biol.

[B23] Zhu L, Wilken J, Phillips NB, Narendra U, Chan G, Stratton SM, Kent SB, Weiss MA (2000). Sexual dimorphism in diverse metazoans is regulated by a novel class of intertwined zinc fingers. Genes Dev.

[B24] Erdman SE, Burtis KC (1993). The Drosophila doublesex proteins share a novel zinc finger related DNA binding domain. Embo J.

[B25] Raymond CS, Shamu CE, Shen MM, Seifert KJ, Hirsch B, Hodgkin J, Zarkower D (1998). Evidence for evolutionary conservation of sex-determining genes. Nature.

[B26] Yi W, Zarkower D (1999). Similarity of DNA binding and transcriptional regulation by Caenorhabditis elegans MAB-3 and Drosophila melanogaster DSX suggests conservation of sex determining mechanisms. Development.

[B27] Ross JM, Kalis AK, Murphy MW, Zarkower D (2005). The DM domain protein MAB-3 promotes sex-specific neurogenesis in C. elegans by regulating bHLH proteins. Dev Cell.

[B28] An W, Cho S, Ishii H, Wensink PC (1996). Sex-specific and non-sex-specific oligomerization domains in both of the doublesex transcription factors from *Drosophila melanogaster*. Mol Cell Biol.

[B29] Erdman SE, Chen HJ, Burtis KC (1996). Functional and genetic characterization of the oligomerization and DNA binding properties of the Drosophila doublesex proteins. Genetics.

[B30] Cho S, Wensink PC (1996). Purification and physical properties of the male and female double sex proteins of Drosophila. Proc Natl Acad Sci U S A.

[B31] Narendra U, Zhu L, Li B, Wilken J, Weiss MA (2002). Sex-specific gene regulation. The Doublesex DM motif is a bipartite DNA-binding domain. J Biol Chem.

[B32] Muller CW, Herrmann BG (1997). Crystallographic structure of the T domain-DNA complex of the Brachyury transcription factor. Nature.

[B33] An W, Wensink PC (1995). Integrating sex- and tissue-specific regulation within a single Drosophila enhancer. Genes Dev.

[B34] Pollock R, Treisman R (1990). A sensitive method for the determination of protein-DNA binding specificities. Nucleic Acids Res.

[B35] Thompson W, Rouchka EC, Lawrence CE (2003). Gibbs Recursive Sampler: finding transcription factor binding sites. Nucleic Acids Res.

[B36] Thompson W, Palumbo MJ, Wasserman WW, Liu JS, Lawrence CE (2004). Decoding human regulatory circuits. Genome Res.

[B37] Kim S, Kettlewell JR, Anderson RC, Bardwell VJ, Zarkower D (2003). Sexually dimorphic expression of multiple doublesex-related genes in the embryonic mouse gonad. Gene Expr Patterns.

[B38] Zhang W, Li B, Singh R, Narendra U, Zhu L, Weiss MA (2006). Regulation of sexual dimorphism: mutational and chemogenetic analysis of the doublesex DM domain. Mol Cell Biol.

[B39] Coschigano KT, Wensink PC (1993). Sex-specific transcriptional regulation by the male and female doublesex proteins of Drosophila. Genes Dev.

[B40] Dalton S, Treisman R (1992). Characterization of SAP-1, a protein recruited by serum response factor to the c-fos serum response element. Cell.

[B41] Sampler GM http://bayesweb.wadsworth.org/cgi-bin/gibbs.9.pl?data_type=DNA.

[B42] Weblogo http://weblogo.berkeley.edu/logo.cgi.

[B43] Huynh KD, Fischle W, Verdin E, Bardwell VJ (2000). BCoR, a novel corepressor involved in BCL-6 repression. Genes Dev.

[B44] Crooks GE, Hon G, Chandonia JM, Brenner SE (2004). WebLogo: a sequence logo generator. Genome Res.

[B45] Schneider TD, Stephens RM (1990). Sequence logos: a new way to display consensus sequences. Nucleic Acids Res.

