# Supplementary material for: Vertebrate DM domain proteins bind similar DNA sequences and can heterodimerize on DNA
Source: BMC Mol Biol. 2007 Jul 2;8:58. doi: 10.1186/1471-2199-8-58 (PMC1931443; doi:10.1186/1471-2199-8-58)
Supplement: Additional file 1 — Sequences of selected sites. This file includes all of the selected sequences used to determine the described binding consensus sites. [file 1471-2199-8-58-S1.doc]

**Murphy et al Supplementary Figure 1**

Sequences of selected sites encompassing the consensus for the indicated DM domain proteins.

Uppercase letters indicate consensus sequences found by Gibbs Motif Sampler. Lower case extensions of sequences were included to generate consistent length Weblogo representations for Figure 2a.

**DSXF sites (From Yi and Zarkower 1999)**

aataaCGCAACATTGTAGc

agcggCTCTACAAAGTATa

cgcggTGCAACTTTGTTGc

actacAGCTACAATGTATg

cctgtCGCTAGATTGTAGc

ctgtcGGCTACTTTGTAGt

tgactTGACACATTGTATg

tgtcgTGCAACTTTGTATt

cctcgTACAACAAAGTATc

gacgtTAATACAATGTATa

ctcccTACAACTTTGTAAg

atctgTGTAACAATGTTGt

ggattTACAACAAAGTAAg

atccaTGCAACTATGTAAc

ctaaaTGCTACTTTGTAAc

gagtgTGAAACATTGTATg

aaattCATCACATTGTATt

accagTGAAACATTGTAAg

tcggtCGTTACATTGTAAg

ataggGGTTACATTGTATg

gcctcTGCAAGAATGTAGc

cacgcTGCAACTATGTAGc

tctggCATTACAATGTAAa

tctaaAACAACATAGTAAg

ctacgTGCAACATTGTAAt

cctgtCGAGACATTGTATc

gtcggTGCAACATAGTAGt

tcgttCTATACTTTGTATg

tcctgTGCAACAATGTGTc

tccaaCGCAACAATGTATg

tggtgTAATACTTTGTGTt

tgaacAGTTACAATGAAAg

atcgcAATTACATTGTAAc

ggtgaTGCAACTATGTTGc

cctgtCGCAACATTGTATg

cccttGACGACAATGTATa

ctcccATCAACAATGTAAg

cgggtTGCAACAATGTTGt

tattaGACGACAATGTATc

gcgacTGGTACATTGTAGg

aaccgTACAACAAGGTAGt

cctgtAGTGACATTGTAGc

cgtggTGCAACAAAGTAGt

cgcctCGCTACAATGTAAg

acacgAACAACAATGTATg

ccttaCATTACATTGTGTg

cgctaCGATACATTGTATg

gtcgcCCGTAGATTGTATg

cgaagGACAACAAAGTATc

caacaAACAACATTGTAAg

cctgtCGCAACTTTGTGGt

agagaTGCAACTTTGTTGt

atcctGTCGACATTGTATt

accctTGTTACTTTGTATc

tggttTGCAACGTTGTATc

accagTAACACATTGTATa

accacTGCAACAATGTTAa

**DMRT1**

gataTTGCAACATTGTTTA

ctaaCTGATACATTGTTTC

ctaaTTGATACTTTGTGTT

aaagTGGCAACAATGTTGC

tgttTTGATACATTGTTGA

tggtTAGTTACAATGTTGC

ggatATGCTACATTGTTGC

ccaaTTGCTACAATGTTGC

atatTTGATACAATGTATG

aatgTTGATACATTGTAAC

tctaATGTTACATTGTTGC

ataaTTGCAACAATGTATT

aaaaCTGATACAATTCAGT

gaacTTGCAACAATGTTGC

acttTAGATACATTGTATT

tcagCGGCAACATTGTTGC

ggagTTGTAACAATGTTTG

gagtTGGTTACATTGTATC

ctgtCGGATACAATGTTTC

tgccTGGCGACATTGTTGC

gtccCTGATACATTGTTGC

cgtgCTGATACATTGTTGC

**DMRT2**

GAAATTGATACATTGTagc

AGATTTGATACATTGTtgc

CAAAATGATACATTTAgag

TAAATTGATACAATTAaac

TAAATTGATACAATGTcct

CAATAGAATCCAATGAccg

CATATTGATACAATGTatg

CAGAGTGATACATTGTatc

GATATTGCTACATTGTtta

CAATGTGTTACATTGAgag

TGGTTGGCTACATTGTtgc

GAAATTGATACATTCAcga

GATATTGCAACATTCTaac

TGGTTAGTTACAATGTtgt

CAAAATGATACATTTAacc

GTTATTGTTACATTTTgtg

TATTTTGTTACATTGTatc

CATAATGTTACTTTTTaga

**DMRT3**

tAATTTGTAACATTTTtgg

aAATTTGATACATTGCtac

aATTTTGTTACTTTGTttc

tCGGTGGTTACATTGTtac

gTGTTTGTTGCATTTGagg

cAAGTTGTTACATATGtaa

gGATTCGTTACAATGTgtc

tGGTTGGCTACATTGTtgc

gGTTTTGATACAATTTtac

gAAATTGATACATTCAcga

tCTATTGATACAAAGTatc

aTTATTGATACAATGTgtg

aGTTTGGATACATTTGagg

tAATTTGTTACATTTTtga

gTGTTCGATACATTGTaac

cGATTTGATACAATGTgtg

aAGGTTGTTACAAACTtaa

cATATTGCTACAATATtgc

gGAATCGATACAATTTgta

cACTTTGTTACATTGCgag

cGAACCGTTACATTTCgtt

cATATTGATACATAACatt

cGTTTTGATACAAATTctc

cTAAATGATACATTCAaca

gAAATTGATACAAATAgaa

tCGATTGATACATGTTgct

cAATAGGATACAATGTatc

gCTTTTGATACATTTTgag

tAAATAGATACAATACcaa

gAAATTGATACATTGTagc

cGTTTTGTTACATCGAggc

aCAATTGTTACAATATccg

tAGTTTATTACATTTTcga

cTAATTGTTACATTTAtaa

aTATTTGTTACATGTAgcg

cAATTTGTTACAATACcta

gTTTTTGTTACTTTGTgtc

cAACTTGATACATTCTtca

cGTATTGATACTTTTCatt

tGAGTTGTTACATTTTcag

**DMRT4**

cgcAATGTTACAATGTtgt

ccaAATGTATCAAAATaaa

caaACTGTAACAATTTact

ggaGTTGTTACAAATTgtc

gaaATTGTAACAAAAAccg

ggcAATGTAACAAATAagt

ttgAATGTAGCATTATaat

tgaAATGTAACAATTCgac

ccaAATGTAACAAACCaac

tggGTAGTGGCAGTGCtct

tacATTGTATCGAATAtat

gaaAATGTAACAAAATttc

taaGCTGAAGCGGGGTatc

atcAATGTAACAATTTgag

cgtAATGTAACAGTTGagg

gttAATGTTGCAAGTTgat

cttAATGTTTCGAGTTtgg

tacAAAGTATCAACTTgta

actAATGTAACGAATTagc

catACTGTATCAATTCggt

cgaGTAGTGTGAGTTAggg

gtcAATGTTGCAGTTTtga

tcgACAGTAGCGATGAaga

cttAATGTAACAAATCtcg

ttaGATGTTGCAATTTttg

aaaATAGTTGCAAAACgac

atgCATGTAGGGTATGtcg

tcaAATGTTGCAATATaac

agcAATGTAACGACTTgga

caaAAAGTAACAATAAaca

aggCCTTTATCAGCTAggt

gctTTTGTTGCAATTTtag

tacAATGTATCAGTGTccc

tacAATGTAACAAAATaac

acaAATGTTGCAAATTcaa

caaAAAGTTTCTCGGTtga

ccaAATGTATCAAAATaaa

gtgAATGTATCAATTTcgt

acaTATGTAACAACTTgac

atcTAAGTGGCATTGAgcc

**DMRT5**

cgAAGAGTTACAGTATCgc

ggTTTTGTAACAGTTTCtt

tgTATTGTAACCTTGTAac

taAGTTGTTAGTGTTTCgg

gaAGTTGTTACTTAGTTgc

ccGATTGTTACTGTTTCga

gtTTTTGTTTCAATGTTgc

ccGGTTGTTACATTGTAta

ttATTTGTTACTGAGGCga

tcGTTCGTTACTGTTTCaa

caAATAGTTACAGTTGCaa

gaAATTGTTTCATATTCtc

tcGTCCGATACATTGTAtc

gaTATTGATACTGTGTCta

ttCATTGTAACAGTTGCga

tgTCTTGATACTTTGTAtc

caTAGTGTTACTGTATCtt

ccTCTAGTAACAGTATCca

cgTTTTGTTACTTTGTAtc

gaTTCCGTTACAATGTTgc

gtGATAGATACATTGTGtc

gcAATTGTTACAATGTTac

acTTTTGTTACAAAGTCaa

cgAAAAGTTACAGTTGCgt

gtGTGTGTTACATTGTTgc

taTGCTGTTACTGTATCgt

gaACTTGATTCTGTTGCaa

ttTCTTGTTACTGTTGCga

taATTTGCAAGTGTTGCaa

ttTTTTGATACATTGTAtc

gaTACTGTTACATTTTCtg

gtGTTTGCAACATTGTAta

acATTCGTAACAATTGCag

ccTGTCGTAGCCGTGTCta

agGTTTGTTACAATGAGgc

tgAATTGTAACAGTAACcg

ttTTTGGTTACTGTATTgt

taGTTTGTATCATTTGCga

cgATTTGTAACAATTTCac

ttGATTGATACTGTTGCga

gaAGTCGTAACTGTTGCta

tcGATTGATACAGGTGTag

atTTATGTTACTGTTACat

cgCTTTGCAACAGTTTCtt

ctTTTGGCGACAGAGGCga

gaTTCTGTTACATTGTAgc

gaGGCGGTAACTGTATCac

taTATTGATACTTTGTAgc

gtTAATGTGACTGTTTCta

**DMRT7**

gataTTGCTACATTGTTTa

gtcgATGTAACTACTTCGg

gtgaATGTATCAATTTCGt

tacaTTGTTGCAATTATGt

ttaaTTGTATCAATTTATg

ttaaTCGTTACATTATTGa

caaaTTGATACAATGTATc

gataTTGCTACATTGTTTa

atatTTGATACAATGTATg

caaaTTGCTACTTTTCTCc

gttaAGGACAGCACTTTGc

gtttTGGATACATTGTATt

ggttTTGTTATATCGAGGc

ctggTTGTCAGTGTGGGGg

caaaTTGCTACAGTGTTGc

taatTTGTTACATTTTTGa

tgttTTGCTACATTGCGAg

cgatTTGTATCAATTCGGa

agtcTAGTAGCAATATTTg

cataTGGTCATGGTTTTTg

gcagTTGTTACATTGTGGa

gtcgTGGTTACATCGGGGt

tcggTTTTGTCAGTTTTGc

gagaTAGTAACCTTTTTGg

tcggTGGTTACATTGTTAc

tggtTAGTTACAATGTTGc

tcgtTTGTGGTCGTTTGGt

gaggATGACGCAGTGTTGg

gggcCTGTTGGAGTGAGGc

ggtaTGGCATTAAGTCTGc
